# Risk of major labour-related complications for pregnancies progressing to 42 weeks or beyond

**DOI:** 10.1186/s12916-021-01988-5

**Published:** 2021-05-25

**Authors:** Anthea C. Lindquist, Roxanne M. Hastie, Richard J. Hiscock, Natasha L. Pritchard, Susan P. Walker, Stephen Tong

**Affiliations:** 1 Department of Obstetrics and Gynaecology, University of Melbourne, Melbourne, Victoria Australia; 2 Mercy Perinatal, Mercy Hospital for Women, 163 Studley Rd., Heidelberg, Victoria 3084 Australia

**Keywords:** Obstetric, Relative risk, Perinatal, Labour, Macrosomia, Shoulder dystocia, Post-partum haemorrhage, Caesarean section

## Abstract

**Background:**

Post-term gestation beyond 41^+6^ completed weeks of gestation is known to be associated with a sharp increase in the risk of stillbirth and perinatal mortality. However, the risk of common adverse outcomes related to labour, such as shoulder dystocia and post-partum haemorrhage for those delivering at this advanced gestation, remains poorly characterised. The objective of this study was to examine the risk of adverse, labour-related outcomes for women progressing to 42 weeks gestation or beyond, compared with those giving birth at 39 completed weeks.

**Methods:**

We performed a state-wide cohort study using routinely collected perinatal data in Australia. Comparing the two gestation cohorts, we examined the adjusted relative risk of clinically significant labour-related adverse outcomes, including macrosomia (≥ 4500 at birth), post-partum haemorrhage (≥1000 ml), shoulder dystocia, 3rd or 4th degree perineal tear and unplanned caesarean section. Parity, maternal age and mode of birth were adjusted for using logistic regression.

**Results:**

The study cohort included 91,314 women who birthed at 39 completed weeks and 4317 at ≥42 completed weeks. Compared to 39 weeks gestation, those giving birth ≥42 weeks gestation had an adjusted relative risk (aRR) of 1.85 (95% CI 1.55–2.20) for post-partum haemorrhage following vaginal birth, 2.29 (95% CI 1.89–2.78) following instrumental birth and 1.44 (95% CI 1.17–1.78) following emergency caesarean section; 1.43 (95% CI 1.16–1.77) for shoulder dystocia (for non-macrosomic babies); and 1.22 (95% CI 1.03–1.45) for 3rd or 4th degree perineal tear (all women). The adjusted relative risk of giving birth to a macrosomic baby was 10.19 (95% CI 8.26–12.57) among nulliparous women and 4.71 (95% CI 3.90–5.68) among multiparous women. The risk of unplanned caesarean section was 1.96 (95% CI 1.86–2.06) following any labour and 1.47 (95% CI 1.38–1.56) following induction of labour.

**Conclusions:**

Giving birth at ≥42 weeks gestation may be an under-recognised risk factor for several important, labour-related adverse outcomes. Clinicians should be aware that labour at this advanced gestation incurs a higher risk of adverse outcomes. In addition to known perinatal risks, the risk of obstetric complications should be considered in the counselling of women labouring at post-term gestation.

## Background

Post-term gestation is defined as a pregnancy advancing beyond 41^+6^ completed weeks of gestation [1]. The sharp increase in the risk of stillbirth and perinatal mortality beyond this gestation is well-established [1, 2]. As a consequence, post-term induction of labour by 41^+6^ weeks is routinely recommended in many Western jurisdictions. The NICE guidelines in the UK state that induction of labour should be recommended to women with uncomplicated pregnancies between 41^+0^ and 42^+0^ weeks [3], the WHO recommends induction of labour for any women who have reached 41+0 weeks [4] and the Australian and New Zealand College of Obstetricians and Gynaecologists advises that women be counselled about induction of labour after 41 weeks [5], in line with both NICE [3] and the American College of Obstetricians and Gynaecologists guidelines [6]. However, the risk of common adverse outcomes related to labour, such as macrosomia, shoulder dystocia and post-partum haemorrhage for those delivering at this advanced gestation, remains poorly characterised.

The US-based ARRIVE trial, published in 2018, randomised 6000 low-risk nulliparous women to induction of labour or expectant management at 39 weeks gestation. This landmark trial demonstrated a reduced risk of caesarean section and severe perinatal complications following elective induction of labour in low-risk women at 39 weeks and thus provided reassurance to many women and clinicians about the safety of elective delivery at this gestation [7]. Many women still opt to await spontaneous labour beyond their expected date of birth. For women who continue their pregnancy, approximately 7% will progress beyond 41^+6^ weeks of pregnancy without labouring [8]. Given the assurances of the ARRIVE trial, offering an induction of labour is a viable alternative management pathway that is unlikely to increase the risk of complications. It may therefore be informative to both clinicians and patients to characterise the degree of risk for significant labour complications for those who opt to wait 2 weeks beyond their expected date of birth, in the hope of avoiding an induction and undergoing spontaneous labour.

Our study compared labour-related outcomes among women who gave birth at 42 completed weeks and beyond (42^+0^–43^+6^) with those who gave birth at 39 completed weeks (39^+0^–39^+6^). The aim of this study was to determine labour-related risks of progressing to 42 weeks of pregnancy or beyond, compared with those giving birth at 39 completed weeks of pregnancy. Thirty-nine weeks completed gestation was chosen as a reference due to the evidence of favourable perinatal outcomes at this gestation and the findings of the ARRIVE trial which identified this as a viable birth choice that does not increase obstetric risk and avoids the risk of progressing to a significantly advanced gestation undelivered [7].

## Methods

We performed a retrospective cohort study of 95,631 women who gave birth in the Australian state of Victoria between 2009 and 2014. Validated data were obtained from the Consultative Council on Obstetric and Paediatric Mortality and Morbidity (CCOPMM) for all births in Victoria [9–11]. CCOPMM is the central agency that collects data on obstetric and perinatal outcomes within the state. These data are populated by state-wide hospital documentation of outcomes by the midwives caring for the patients.

We examined singleton pregnancies that reached term gestation (≥37^+0^ weeks) and delivered a liveborn neonate between 1st Jan 2009 and 31st Dec 2014. Both comparison groups included parous and nulliparous women, spontaneous and induced labours, spontaneous and assisted vaginal birth and unplanned, emergency caesarean section (elective ceasarean sections were excluded). Given the structure of the dataset, it was not possible to identify maternal clusters (children born to the same mother). Cases were excluded if they had missing or incomplete gestational age data. Completed week of gestational age was the primary exposure in this analysis with gestation in days calculated using the date of birth of the baby relative to the estimated due date.

The outcomes we selected to investigate were major adverse labour-related complications with clinical relevance. These were macrosomia ≥4500g at birth, post-partum haemorrhage (documented as ≥1000ml, typically a weighed estimate), shoulder dystocia (formally documented following the birth) or obstetric anal sphincter injury (documented 3rd or 4th degree tear) following vaginal birth. We also examined the risk of unplanned (emergency) caesarean section following any labour and following induction of labour.

The distribution of covariates between exposures, gestational age at birth (39 vs ≥42 completed weeks), was examined using univariable logistic regression for categorical data and non-parametric Wilcoxon rank-sum test for continuous data. Potential confounding variables were selected based on a priori evidence of likely confounding between these variables and the outcomes examined, and the results of univariate analysis demonstrating significant variation between the gestation cohorts. Potential confounders included in the regression model to produce adjusted risk estimates for each outcome of interest were parity (defined as the number of previous births over 20 weeks gestation and modelled as binary (nulliparous/multiparous)), maternal age (recorded to the nearest year at prenatal booking and centred at 30 years) and mode of birth (as a three-level nominal variable: normal vaginal birth, instrumental vaginal birth and emergency caesarean section).

Interactions were assessed between exposure (gestational age 39 vs 42 weeks) and all covariates included in the adjusted models. Where an interaction was identified, stratified risk measures were determined and presented accordingly. Unadjusted and adjusted relative risk (RR) and 95% confidence intervals (95% CI) were computed using predicted probabilities derived from logistic regression, with 95% CI calculated using natural logarithms and back transformed to the standard metric [12]. Adjusted risk difference (RD) and 95% CI were computed using ordinary least squares linear regression with robust standard errors [13].

Statistical analysis was performed using Stata v15 statistical software. The two-sided significance level was set at 0.05 and not adjusted for multiple comparisons. An a priori decision was made not to impute missing outcome data unless outcome missingness was different across exposure status and to only perform multiple imputation of covariates used in adjusted analyses if missingness exceeded 1%. By design, missingness did not occur for the exposure, gestational age at birth (39 vs ≥42 completed weeks).

At a two-sided significance level of 0.05 and a referent rate of 5%, this study has a power greater than 90% to detect a RD of 1% or a RR of 1.22 given a cohort size of 100,000 and 5000 in exposure arms and a RD of 1.75% or RR of 1.41 for outcomes with 20,000 and 1500 in comparison arms.

## Results

**Fig. 1:**
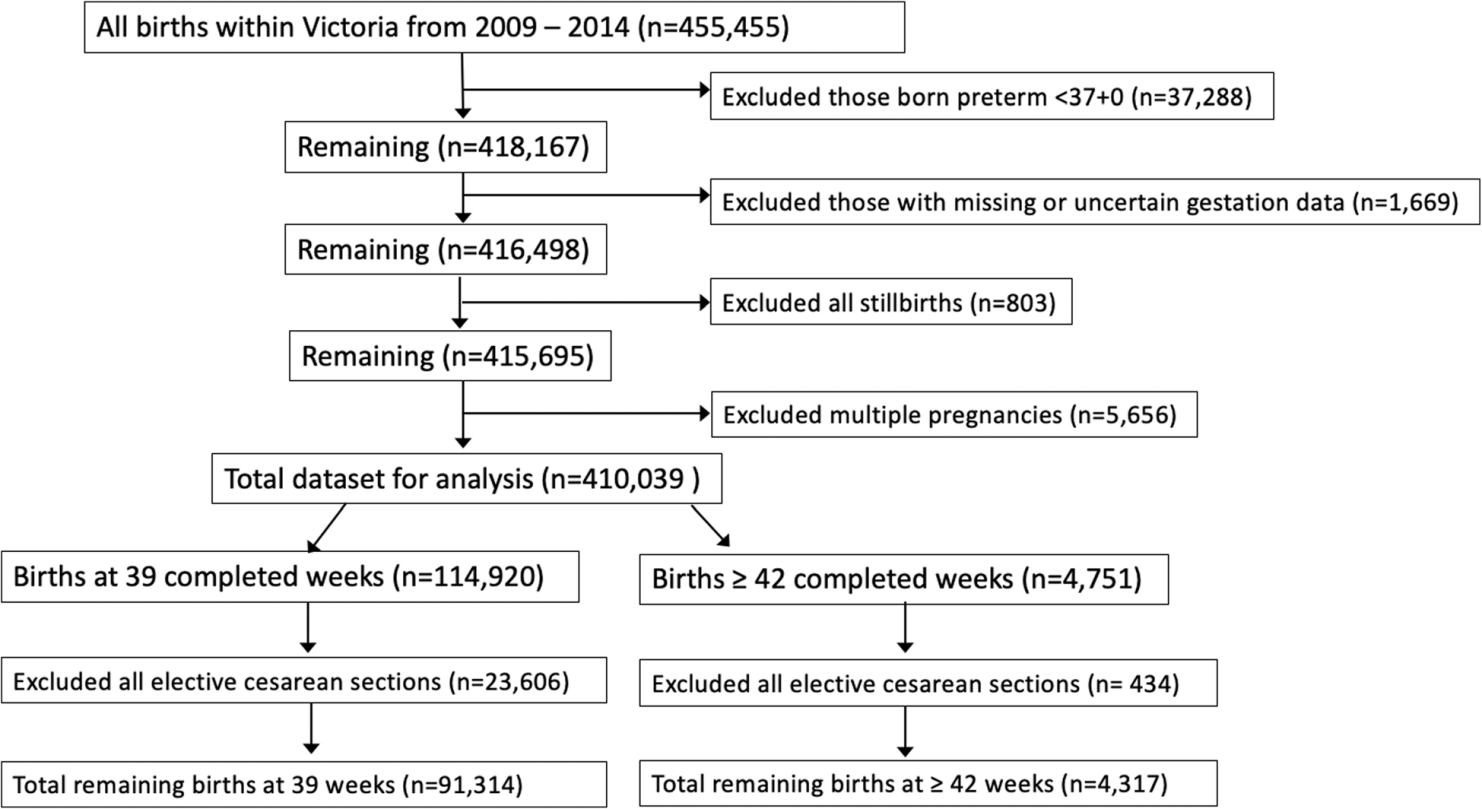
Flowchart of study cohort and exclusions

Following exclusions, the study cohort included 332,413 births during the period 2009–2014. This comprised 91,314 births at 39 completed weeks and 4317 at ≥42 completed weeks (Fig. 1). Figure 1 depicts the number of births excluded by each criterion.

Table 1 shows the clinical characteristics of the two gestation cohorts. Pairwise comparisons indicated that women giving birth at ≥42 weeks were more likely to be nulliparous (58.9 vs 45.5%, *p*<0.001) or grand-multiparous (2.5 vs 1.2%, *p*<0.001). They were also more likely to have an emergency, unplanned caesarean section (26.5 vs 11.8%, *p*<0.001) than a vaginal birth. Missingness for covariates was minimal: parity (0.02%), mode of birth (0.03%) and maternal age (0.08%), and therefore, covariate imputation was not used. Based on the whole cohort, missingness was low for emergency caesarean section (0%), shoulder dystocia (0%), macrosomia (0.14%) and post-partum haemorrage (0.8%), whilst for those who delivered vaginally, missingness was slightly higher for 3rd or 4th degree perineal tear (7.6%). There was no clinically important difference between missing outcomes among the two exposure cohorts.

**Table 1:** Participant characteristics (excluding women who had an elective caesarean)

Characteristics	39 completed weeks ***n*** (%) (***N*** = 91,314)	≥42 completed weeks ***n*** (%) (***N*** = 4317)	***p***
**Parity**^a^			
Nulliparous (0 previous births)	41,513 (45.5)	2543 (58.9)	<0.001
Multiparous (1–4 previous births)	48,656 (53.3)	1667 (38.6)	
Grand-multiparous (≥5 previous births)	1124 (1.2)	106 (2.5)	
**Maternal age**^a^			
Mean (SD)	30.3 years (5.30)	30.3 years (5.29)	0.66
**Maternal age**^a^			
13–24	13,343 (14.6)	633 (14.7)	0.91
25–34 (baseline)	58,139 (63.7)	2731 (63.3)	
35–44	19,648 (21.5)	944 (21.9)	
≥45	105 (0.1)	6 (0.1)	
**Labour**^a^			
Spontaneous	46,310 (50.7)	982 (22.8)	<0.001
Induced	22,172 (24.3)	2661 (61.6)	
Augmented	22,811 (25.0)	674 (15.6)	
**Mode of birth**^a^			
Unassisted vaginal birth	64,399 (70.5)	2328 (53.9)	<0.001
Assisted (instrumental) vaginal birth	16,151 (17.7)	846 (19.6)	
Emergency caesarean	10,733 (11.8)	1142 (26.5)	

Data presented as mean (SD) or number (%) based upon non-missing values

^a^There were <1% data missing across parity, maternal age, labour and mode of birth

Compared to those giving birth at 39 weeks, women who gave birth at ≥42 weeks had higher rates of macrosomia at birth (5.4 vs 0.5% for nulliparous women and 6.9 vs 1.4% for multiparous women, *p*<0.001), post-partum haemorrhage across all modes of birth (5.5 vs 2.9% for normal vaginal birth, 12.2 vs 5.3% for instrumental vaginal birth and 8.4 vs 5.7% for emergency caesarean section; *p*<0.001), shoulder dystocia (for non-macrosomic babies; 2.9 vs 2.0%, *p*<0.001) and 3rd or 4th degree tear (4.2 vs 2.9%, *p*<0.001) (Table 2). The rate of unplanned ceasarean section was also higher for women giving birth at ≥42 weeks following any labour (26.5 vs 11.8%, *p*<0.001) and following induction of labour (31.8 vs 18.9%, *p*<0.001) (Table 2).

**Table 2:** Unadjusted and adjusted relative risk and risk difference estimates for outcomes of interest (*N*=95,631)

Outcome	39 completed weeks ***n*** (%) (***N***=91,314)	≥42 completed weeks ***n*** (%) (***N***=4317)	Unadjusted relative risk (95% CI) ***p***-value	Adjusted relative risk (95% CI)	Adjusted risk difference (95% CI) ***p***-value
**Macrosomia ≥4500g at birth—nulliparous**					
No	41,247 (99.4)	2404 (94.5)	10.35 (8.39–12.77) <0.001	10.19^a^ (8.26–12.57) < 0.001	4.9%^a^ (4.0–5.7) < 0.001
Yes	216 (0.5)	137 (5.4)			
*Missing*	50 (0.1)	2 (0.1)			
**Macrosomia ≥4500g at birth—multiparous**					
No	48,983 (98.4)	1648 (93.0)	4.80 (3.98–5.78) <0.001	4.71^a^ (3.90–5.68) < 0.001	5.3%^a^ (4.2–6.5) < 0.001
Yes	714 (1.4)	122 (6.9)			
*Missing*	83 (0.2)	3 (0.2)			
**Post-partum haemorrhage ≥1000ml—normal vaginal birth**					
Blood loss <1000ml	62,253 (96.7)	2194 (94.2)	1.87 (1.57–2.23) <0.001	1.85^b^ (1.55–2.20) < 0.001	2.5%^b^ (1.6–3.5) < 0.001
Blood loss ≥1000ml	1887 (2.9)	128 (5.5)			
*Missing*	259 (0.4)	6 (0.3)			
**Post-partum haemorrhage ≥1000ml—instrumental vaginal birth**					
Blood loss <1000ml	15,216 (94.2)	737 (87.1)	2.30 (1.90–2.79) <0.001	2.29^b^ (1.89–2.78) < 0.001	6.9%^b^ (4.7–9.1) < 0.001
Blood loss ≥1000ml	857 (5.3)	103 (12.2)			
*Missing*	78 (0.5)	6 (0.7)			
**Post-partum haemorrhage ≥1000ml—emergency caesarean section**					
Blood loss <1000ml	9746 (90.8)	1011 (88.5)	1.46 (1.19–1.80) <0.001	1.44^b^ (1.17–1.78) 0.001	2.7%^b^ (1.0–4.4.4) 0.002
Blood loss ≥1000ml	612 (5.7)	96 (8.4)			
*Missing*	375 (3.5)	35 (3.1)			
**Shoulder dystocia (vaginal birth only, non-macrosomic babies)**					
No	78,147 (98.0%)	2907 (97.1%)	1.27 (1.03–1.57) < 0.02	1.43^b^ (1.16–1.77) 0.001	0.9%^b^ (0.3–1.4) 0.005
Yes	1567 (2.0%)	87 (2.9%)			
**Shoulder dystocia (vaginal birth only, macrosomic babies)**					
No	582 (77.6%)	146 (83.0%)	0.63 (0.44–0.91) 0.01	0.70^b^ (0.49–1.01) 0.06	−5.5%^b^ (−11.8 to 0.9) 0.09
Yes	168 (22.4%)	30 (17.9%)			
**3rd or 4th degree tear (all women, vaginal birth only)**					
Nil	71,218 (88.4)	2788 (87.8)	1.45 (1.22–1.72) <0.001	1.22^c^ (1.03–1.45) 0.02	0.9%^c^ (0.1–1.6) 0.02
3rd/4th degree tear	2325 (2.9)	134 (4.2)			
*Missing*	7007 (8.7)	252 (7.9)			
**Unplanned caesarean (elective caesarean excluded) following any labour**					
Vaginal birth	80,550 (88.2)	3174 (73.5)	2.25 (2.13–2.37) <0.001	1.96^a^ (1.86–2.06) <0.001	12.7%^a^ (11.4–14.0) < 0.001
Emergency caesarean	10,733 (11.8)	1142 (26.5)			
*Missing*	31 (0.03)	1 (0.02)			
**Unplanned caesarean (elective caesarean excluded) following induced labour**					
Vaginal birth	17,963 (81.0)	1814 (68.2)	1.68 (1.58–1.79) <0.001	1.47^a^ (1.38–1.56) < 0.001	9.9%^a^ (8.2–11.7) <0.001
Emergency caesarean	4197 (18.9)	846 (31.8)			
*Missing*	12 (0.05)	1 (0.04)			

^a^Adjusted for parity (nulliparous/multiparous) and maternal age

^b^Adjusted for parity (nulliparous/multiparous), maternal age and mode of birth

^c^Adjusted for parity (nulliparous/multiparous), maternal age, mode of birth and macrosomia

We next determined the relative risk and risk difference of these obstetric outcomes after adjusting for parity, maternal age and mode of birth (Table 2). Interactions were also tested for all adjusted models between the exposure and each covariate. Interactions (defined as *p*-value <0.01 for the interaction term in the unadjusted RR model) were found between gestational age and the following: parity for macrosomia, mode of birth for post-partum haemorrhage and macrosomia for shoulder dystocia (all interaction tests *p* <0.001). Results were therefore stratified by the relevant interaction for these outcomes. There was no evidence for an interaction between gestational age and parity (unadjusted RR model: *p* = 0.57) for 3rd or 4th degree perineal tears.

The adjusted relative risk (aRR) for those giving birth at ≥42 weeks compared with women giving birth at 39 weeks was 10.19 (95% CI 8.26–12.57) for macrosomia among nulliparous women and 4.71 (95% CI 3.90–5.68) among multiparous women; 1.85 (95% CI 1.55–2.20) for post-partum haemorrhage following spontaneous vaginal birth, 2.29 (95% CI 1.89–2.78) following instrumental vaginal birth and 1.44 (95% CI 1.17–1.78) following emergency ceasarean section; 1.43 (95% CI 1.16–1.77) for shoulder dystocia (non-macrosomic babies); and 1.22 (95% CI 1.03–1.45) for 3rd or 4th degree perineal tears (all women). The aRR risk of shoulder dystocia for those giving birth at ≥42 weeks compared with 39 weeks for women with macrosomic babies was RR 0.70 (95% CI 0.49–1.01).

Following any labour, women giving birth at ≥42 weeks had an aRR of unplanned caesarean section of 1.96 (95% CI 1.86–2.06) and a risk difference (excess risk greater than those at 39 weeks) of 12.7% (95% CI 11.4–14.0). Following an induced labour, women giving birth at ≥42 weeks had an aRR of 1.47 (95% CI 1.38–1.56) of unplanned caesarean and a risk difference of 9.9% (95% CI 8.2–11.7) compared with women giving birth at 39 weeks (Table 2).

## Discussion

Our large cohort study demonstrates that women labouring at an advanced post-term gestation are at significantly elevated risk of a number of major adverse labour-related outcomes. The increases appear quite marked across many adverse outcomes: compared with those giving birth at 39 weeks gestation, women giving birth at ≥42 weeks gestation incurred an 8–12-fold increased risk of birthing a macrosomic baby, an approximately 85% increased risk of post-partum haemorrhage, 22% increased risk of major perineal tears (3rd or 4th degree) and a 96% increased risk of an unplanned caesarean section.

For all outcomes, unadjusted and adjusted RR point estimates were similar. The evidence for an interaction between macrosomia and parity, post-partum haemorrhage and mode of birth and shoulder dystocia and macrosomia is evident in the raw stratified tables and was confirmed as highly statistically significant for all metrics.

The risk of shoulder dystocia was increased to 1.43 (95% CI 1.16–1.77) at 42 completed weeks for non-macrosomic babies only, with borderline evidence of a reduced risk for macrosomic babies (RR 0.70; 95% CI 0.49–1.01). It is possible that this represents a lack of power due to smaller numbers of macrosomic babies overall and very small numbers experiencing a shoulder dystocia. However, it is also plausible that babies identified antenatally as macrosomic are delivered earlier than 42 weeks, and only those where the risk of shoulder dystocia for the mother is considered to be low are allowed to progress to advanced gestation.

We examined a large, state-wide cohort in Australia over a 5-year period and selected specific adverse outcomes that are likely to be regarded as significant and impactful by both patients and clinicians. Whilst many risk factors for these common complications have been characterised, post-term gestation is not widely reported as a risk factor for post-partum haemorrhage [14–16], shoulder dystocia (except often in the setting of known macrosomia) [17, 18] or unplanned caesarean section. Our findings suggest that post-term gestation should be considered a significant risk factor for adverse labour outcomes.

Whilst clinicians may have suspected that births ≥42 weeks gestation entail increased risk, the relative magnitude of this increase (>40% for multiple adverse outcomes) is likely to have been under-appreciated in the clinic. Our findings have clinical relevance. Clinicians should be aware that those in labour at 42 weeks gestation and beyond represent a cohort at significant risk for a number of common obstetric complications. Furthermore, knowing that a safe alternative management option exists (induction of labour at 39 weeks gestation [7, 19]), this information is likely to be useful when counselling patients as to the timing of induction of labour for pregnancies that have progressed beyond their due date.

In order to investigate our hypothesis, it was necessary to select a reference gestation to compare the risk of adverse outcomes. We selected 39 weeks gestation for two important reasons: it is the gestation when foetuses are most commonly born and a gestation where there is a justifiable clinical option to induce labour [7]. In light of our significant clinical findings, it will be worthwhile for future studies to determine the incremental risk of these adverse outcomes at 40 and 41 weeks gestation. We anticipate the risk would rise progressively across each week of advancing gestation.

### Strengths and limitations

We believe our data has a number of strengths. The first is the size of the cohort and our capacity to capture data from an entire state. Second, our dataset included very little missing data for the confounders of interest (<0.08%), which meant that imputation techniques to account for missing data were not required for adjusted analysis. The amount of missing outcome data was less than 1%, except for vaginal tears. The magnitude of missing outcome data for obstetric anal sphincter injury is consistent with that reported in the literature. This outcome is notoriously poorly reported [20, 21]. The decision not to impute outcomes, including tears, was made based upon the lack of difference in outcome missingness across exposure cohorts, whereby the results should be unbiased [22].

The limitations include that this was a retrospective study, that we only demonstrate associations not causal effects and that the dataset did not allow for identification of clustering by birth mother which will lead to 95% CI that are too narrow. Standard error underestimation is reduced when the number of clusters is large (our data) and when within-cluster correlation is low (unknown in this setting); however, it can persist even at cluster sizes of 2 to 5 (likely in birth cohorts) [23]. Despite these limitations, we believe our findings are biologically plausible and that the increase in these adverse outcomes could be prevented if these women were delivered at an earlier gestation. The next study of interest would be to assess whether the risk of adverse outcome at post-term gestation can be feasibly mitigated or reduced by planned earlier elective induction of labour.

Within the range of term gestation (37^+0^–40^+6^), there is clearly a balance to be found between the risks and benefits at different gestational ages for both mother and baby. The risk of adverse neonatal outcomes such as neonatal death, respiratory compromise, hypoglycaemia, sepsis and admission to the Neonatal Intensive Care Unit is known to be lowest among uncomplicated pregnancies delivered between 39^+0^ and 40^+6^ weeks of gestation [19, 24, 25]. Furthermore, there is growing evidence about the long-term neurodevelopmental benefits for children born between 40 and 41 weeks (REF GORDON). From a maternal perspective, the ARRIVE trial provided additional reassurance about the safety of elective induction of labour for low-risk women at 39 weeks.

We are not suggesting that our findings should lead to prescriptive advice to women about avoiding pregnancies reaching post-term gestation. And we acknowledge that our cohort, whilst representative of an entire Australian state, is derived from a high-resource setting and our findings and recommendations may not be applicable in low- or middle-income settings where resources are scarce. However, we believe our findings may enhance shared decision-making in settings similar to our own, where women choosing to await spontaneous labour beyond their expected date of birth should be informed of significantly increased risk of many labour-related adverse outcomes should they progress beyond 42 weeks gestation without having given birth.

## Conclusions

It is well-established that for mothers progressing beyond 42 weeks gestation, their babies are at considerably increased risk of stillbirth and perinatal mortality. Our study demonstrates that labour-related risks for these women are also high and should be considered as part of clinical planning and counselling.

## Data Availability

The data that support the findings of this study are available from the Consultative Council on Obstetric and Paediatric Mortality and Morbidity (CCOPMM), but restrictions apply to the availability of these data, which were used under licence/approval for the current study, and so are not publicly available. Data are, however, available from the authors upon reasonable request and with permission of the Board of CCOPMM.
